# Genetic subtypes and phenotypic characteristics of 110 patients with Prader-Willi syndrome

**DOI:** 10.1186/s13052-022-01319-1

**Published:** 2022-07-23

**Authors:** Lu Zhang, Xiaoliang Liu, Yunjing Zhao, Qingyi Wang, Yuanyuan Zhang, Haiming Gao, Bijun Zhang, Wanting Cui, Yanyan Zhao

**Affiliations:** 1 Department of Clinical Genetics, Shengjing Hospital of China Medical University, No 36 Sanhao Street, Heping Ward, Shenyang, 110004 China; 2 Department of Developmental Pediatrics, Shengjing Hospital of China Medical University, Shenyang, China; 3 Center for Computational and Integrative Biology, Massachusetts General Hospital, Boston, MA USA

**Keywords:** Prader-Willi syndrome, Microdeletion, Uniparental disomy, Phenotype

## Abstract

**Background:**

Prader-Willi syndrome (PWS) is a complex disorder caused by impaired paternally expressed genes on chromosome 15q11-q13. Variable findings have been reported about the phenotypic differences among PWS genetic subtypes.

**Methods:**

A total of 110 PWS patients were diagnosed from 8,572 pediatric patients included from July 2013 to December 2021 by MLPA and MS-MLPA assays. Atypical deletions were defined by genomic CNV-sequencing. Maternal uniparental disomy (UPD) was subgrouped by microsatellite genotyping. Clinical data were collected for phenotype-genotype associations. Twenty-one patients received growth hormone (GH) treatment, and the anthropometric and laboratory parameters were evaluated and compared.

**Results:**

Genetically, the 110 patients with PWS included 29 type I deletion, 56 type II deletion, 6 atypical deletion, 11 heterodisomy UPD, and 8 isodisomy UPD. The UPD group had significantly higher maternal age (31.4 ± 3.4 *vs* 27.8 ± 3.8 years), more anxiety (64.29% *vs* 26.09%) and autistic traits (57.14% *vs* 26.09%), and less hypopigmentation (42.11% *vs* 68.24%) and skin picking (42.86% *vs* 71.01%) than the deletion group. The type I deletion group was diagnosed at earlier age (3.7 ± 3.3 *vs* 6.2 ± 3.2 years) and more common in speech delay (95.45% *vs* 63.83%) than the type II. The isodisomy UPD group showed a higher tendency of anxiety (83.33% *vs* 50%) than the heterodisomy. GH treatment for 1 year significantly improved the SDS of height (− 0.43 ± 0.68 *vs* − 1.32 ± 1.19) and IGF-I (− 0.45 ± 0.48 *vs* − 1.97 ± 1.12). No significant changes were found in thyroid function or glucose/lipid metabolism.

**Conclusion:**

We explored the physical, psychological and behavioral phenotype-genotype associations as well as the GH treatment effect on PWS from a large cohort of Chinese pediatric patients. Our data might promote pediatricians' recognition and early diagnosis of PWS.

**Supplementary Information:**

The online version contains supplementary material available at 10.1186/s13052-022-01319-1.

## Background

Prader-Willi syndrome (PWS; OMIM#176,270) is a complex genetic disorder caused by lacking paternal expression of imprinted genes on chromosome 15q11-q13. The estimated prevalence of PWS is 1/10,000–30,000 live births [1]. PWS has several genetic subtypes: paternal deletions (65–75%), maternal uniparental disomy (UPD) (20–30%), imprinting center defects (1–3%) and other rare cases of paternal chromosome 15 translocation (0.1%) [2]. Five common breakpoint regions (BP1-BP5) have been identified along chromosome 15q11-q13 [3]. The paternal deletions of PWS are usually recurrent involving BP1-BP3 (type I) and BP2-BP3 (type II), and sometimes atypical with smaller or larger deletion sizes [4–6]. Maternal UPD happens due to meiosis errors during female gametogenesis: isodisomy is caused by meiosis II error and later monosomy rescue; heterodisomy is caused by meiosis I error without crossover events; and mixed UPD is related to meiosis I error with crossover events [7, 8].

PWS is a multisystemic condition with phenotype changing dramatically with age. Infantile hypotonia is characteristic, demonstrating weak cry, poor suck, and failure to thrive. Male cryptorchidism is frequent owing to hypogonadism [9]. In early childhood, they gradually exhibit excessive appetite and obesity if unrestricted from food [10]. Developmental delay and/or intellectual disability (DD/ID) are presented as delayed motor/language skills and learning problems [1]. Behavior and psychological problems are common including stubbornness, compulsions, temper tantrums, skin picking, anxiety, and autistic traits [11]. Short stature and small hands/feet are caused by growth hormone deficiency [12]. Hypothalamic dysfunction underlies many features including hyperphagia, temperature instability, pituitary hormone deficiency, high pain threshold, and sleep apnea [13]. Dysmorphic faces and hypopigmentation are also common in PWS patients [2]. To date, phenotypic disparities have been reported between the UPD and deletion types of PWS, showing inconsistent findings. The differences within the UPD and deletion subtypes remain less known.

In this study we presented the overall data of 110 cases with PWS diagnosed from 8,572 pediatric patients in Northeast China. The phenotypic characters were evaluated and compared among different genetic subtypes to explore possible associations. The effect of growth hormone (GH) treatment on 21 cases was also evaluated.

## Methods

### Patients

A total of 8,572 patients with DD/ID and/or suspected PWS features were included from July 2013 to December 2021 through outpatients in pediatrics and inpatients in neonatal ward of Shengjing Hospital of China Medical University. The inclusion criteria were: < 70 of development quotient (DQ) and/or intelligent quotient (IQ) for DD/ID; and ≥ 1 item of ① neonatal hypotonia with poor suck and feeding problems, ② male cryptorchidism, ③ short stature with small hands/feet, ④ obesity with hyperphagia and extreme food seeking, for suspected PWS features. Cases with other definitive genetic aetiology (such as Fragile X syndrome, Down syndrome, etc*.*), abnormal neuroimaging and metabolic screening have been excluded. Written informed consents for participation and publication were obtained from a legal guardian. We followed the Declaration of Helsinki, and all protocols were approved by the Ethics Committee of Shengjing Hospital of China Medical University.

### Phenotypic evaluation

Subject demographics were recorded including maternal age, age, sex, height, weight, and BMI. Clinical features were collected including neonatal hypotonia, feeding problems, dysmorphic faces, light-colored hair and skin, sticky saliva, undescended testes, central and/or obstructive sleep apnea, seizures, hyperphagia, delayed developmental milestones and language skills, learning disabilities, psychosocial and behavioral problems, reduced sensitivity to pain, and medical interventions. DQ was assessed using Gesell Developmental Schedules [14]. IQ was assessed in cooperative children using Wechsler Intelligence Scale for Children [15]. Autistic traits were evaluated using Childhood Autism Rating Scale [16]. Laboratory parameters of patients with recombinant human GH treatment for more than one year were collected from the hospital laboratory center, including serum insulin-like growth factor I (IGF-I), serum thyroid stimulating hormone (TSH), free thyroxine (fT4), free triiodothyronine (fT3), fasting glucose and insulin, total triglyceride, total cholesterol, and low density lipoprotein (LDL). Standard deviation scores (SDS) values were calculated for height, weight, BMI, and IGF-I according to reference values for the Chinese population [17–19].

### Multiplex ligation-dependent probe amplification (MLPA) assay

Genomic DNA was isolated from peripheral blood samples using the Blood Genomic DNA Miniprep Kit (Axygen, CA, USA). MLPA P245 kits (MRC-Holland, Amsterdam, the Netherlands) were used to screen common microdeletion syndromes following the manufacturer's instructions. Briefly, DNA was denatured at 98 °C for 5 min and hybridized with the probes at 60 °C for 16 h. Ligation was performed at 54 °C for 15 min, and ligated probes were subsequently amplified by PCR using universal fluorescent primers. The fragments were separated by capillary electrophoresis using the 3730 Genetic Analyzer (Applied Biosystems, CA, USA) and analyzed using Coffalyser software (MRC-Holland).

### Methylation-specific MLPA (MS-MLPA) assay

MS-MLPA ME028 kits (MRC-Holland) were used for the diagnosis of PWS following the manufacturer's instructions. Denaturing and hybridization were the same as the MLPA assay. The hybridized samples underwent ligation with or without methylation-sensitive restriction enzyme *Hha* I at 48 °C for 30 min, and were amplified by PCR following the same procedure as MLPA. The fragments were separated using the 3730 Genetic Analyzer (Applied Biosystems) and assayed with the Coffalyser software (MRC-Holland).

### Genomic CNV-sequencing

Atypical deletions were defined by genomic CNV-sequencing as previously described [20]. Briefly, 50 ng of genomic DNA was fragmented to construct DNA libraries by end filling, adapter ligation, and PCR amplification. The DNA libraries were constructed using library preparation kits (Berry Genomics, China) and sequenced by the NextSeq 500 platform (Illumina, San Diego, CA, USA) to generate about 8 million raw reads with 36 bp in length. All the sequences were aligned to the GRCh7/hg19 genome using the Burrows-Wheeler algorithm. Mapped reads were allocated progressively to the chromosomes, and copy number changes were evaluated by comparing bin counts between all test samples run in the same flow cell.

### Genotyping of microsatellite markers

Cases with maternal UPD underwent genotyping of 10 polymorphic microsatellite markers on chromosome 15q11-q13 by multiplex PCR using fluorescent primers listed in supplementary Table 1. Amplicons were purified (Axygen) and separated by capillary electrophoresis using the 3730 Genetic Analyzer (Applied Biosystems), and analyzed by the GeneMapper 4.1 software (Applied Biosystems).

### Statistical analyses

All statistical analyses were performed in SPSS-V-23.0 (IBM Corporation, USA). Normal data were expressed as means ± SD or number (percentage). The Shapiro–Wilk test was employed to examine continuous variation. Comparisons between two groups were performed by chi-square test, nonparametric Mann–Whitney U test or Student t test accordingly. *P* < 0.05 was considered to indicate statistical significance.

## Results

### Genetic diagnosis of 110 patients with PWS

**Fig. 1:**
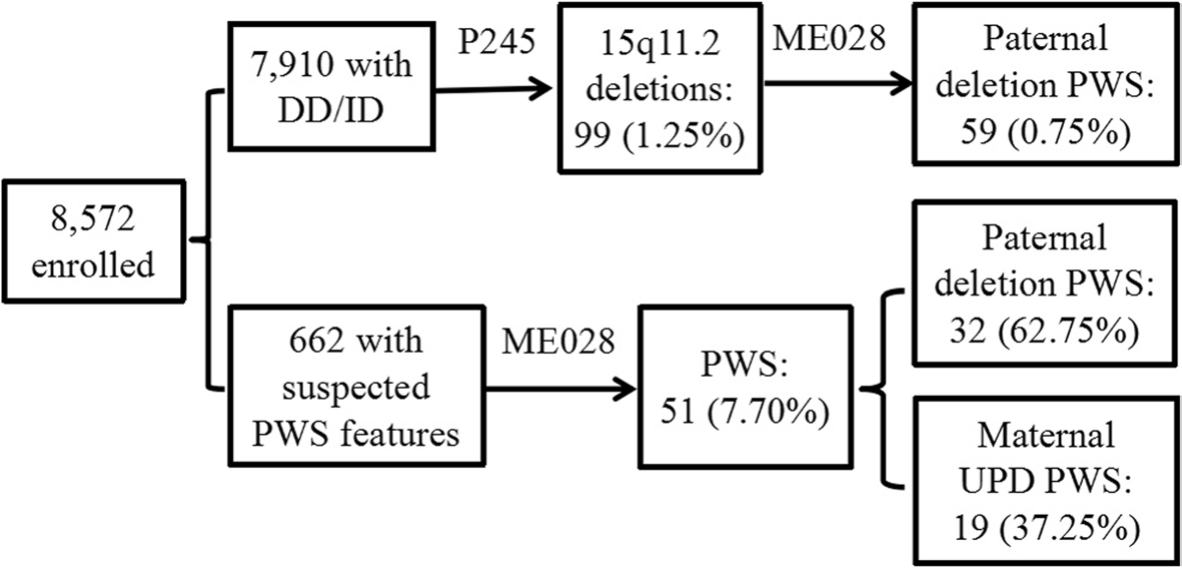
Flow chart of genetic diagnosis of 110 PWS patients from 8,572 pediatric individuals by P245 and ME028 assays

Of the enrolled 8,572 individuals, 7,910 with DD/ID were screened for common microdeletion syndromes by P245, followed by ME028 confirmation on those with 15q11.2 deletions. Ninety-nine (99/7,910, 1.25%) cases were found with 15q11.2 deletions, among which 59 (59/99, 59.60%) with paternal deletions were diagnosed as PWS. The diagnostic yield of paternal deletion PWS was 0.75% (59/7,910) by P245-ME028 assays. The 662 individuals with suspected PWS features were diagnosed directly by ME028, showing a high diagnostic rate of 7.70% (51/662). These 51 PWS patients included 32 (32/51, 62.75%) with paternal deletions and 19 (19/51, 37.25%) with maternal UPD. The collective 110 PWS patients were further assayed for genetic and phenotypic characteristics (Fig. 1).

### Genetic subtypes of 91 patients with paternal deletions

**Fig. 2:**
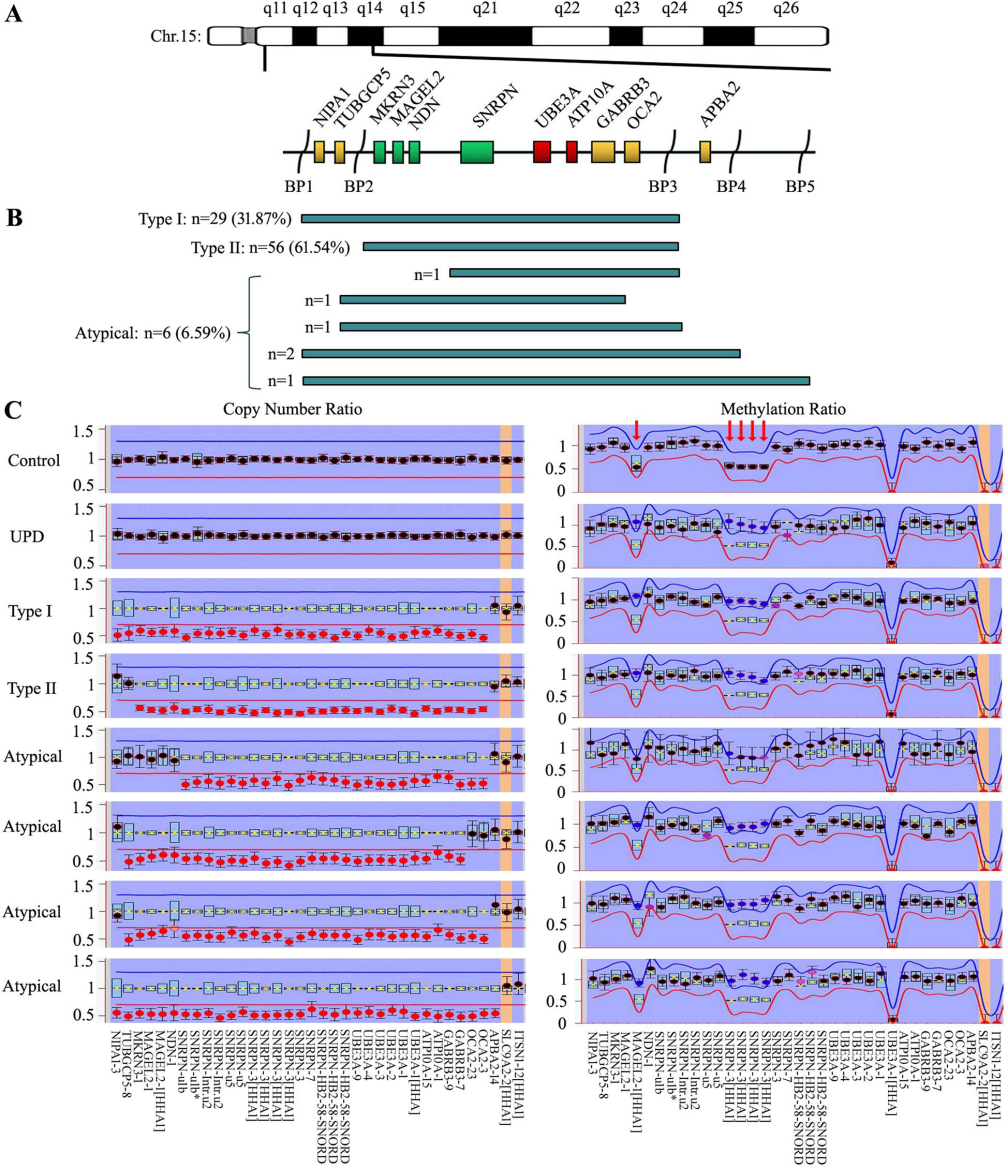
**A** Schematic diagram of ME028 probes for genes on chromosome 15q11-q13. Non-imprinted genes are shown in yellow boxes. Paternally expressed genes are shown in green boxes. Maternally expressed genes are shown in red boxes. **B** Genetic subtypes in 91 PWS patients with paternal deletions. **C** Representative data of the copy number ratio (left panel) and methylation ratio (right panel, red arrows indicated) by ME028 assay

**Fig. 3:**
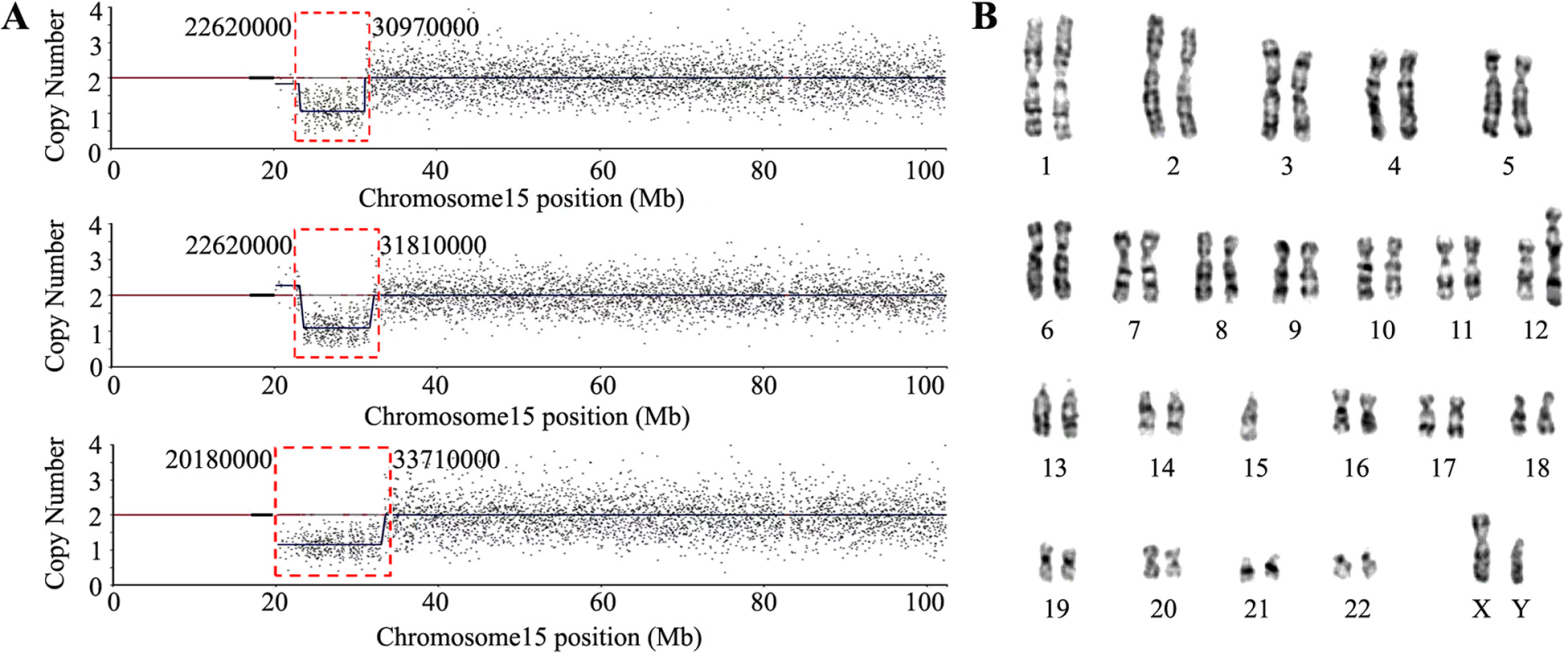
**A** Genomic CNV-sequencing profiles of chromosome 15 in cases with expanded deletions. The blue line indicates the mean copy number. **B** G-band karyotyping of unbalanced translocation of 45,XY,der(12)t(12;15)(q24;q13),–15

ME028 contains dense probes for genes encompassing BP1 to distal BP3 on chromosome 15q11-q13 (schematically presented in Fig. 2A). The 91 patients with paternal deletions were classified into 29 (29/91, 31.87%) type I, 56 (56/91, 61.54%) type II, and 6 (6/91, 6.59%) atypical deletions (Fig. 2B). Representative data of ME028 assay are shown in Fig. 2C. Genomic CNV-sequencing was performed on patients with deletions exceeding the detection range of ME028, showing 2 with expanded deletions to BP4, and 1 with deletion to BP5 (Fig. 3A). All the PWS patients were normal in G-band karyotyping, except for the one with the largest deletion showed chromosomal translocation of 45,XY,der(12)t(12;15)(q24;q13),–15 (Fig. 3B).

### Genetic subtypes of 19 patients with maternal UPD

**Fig. 4:**
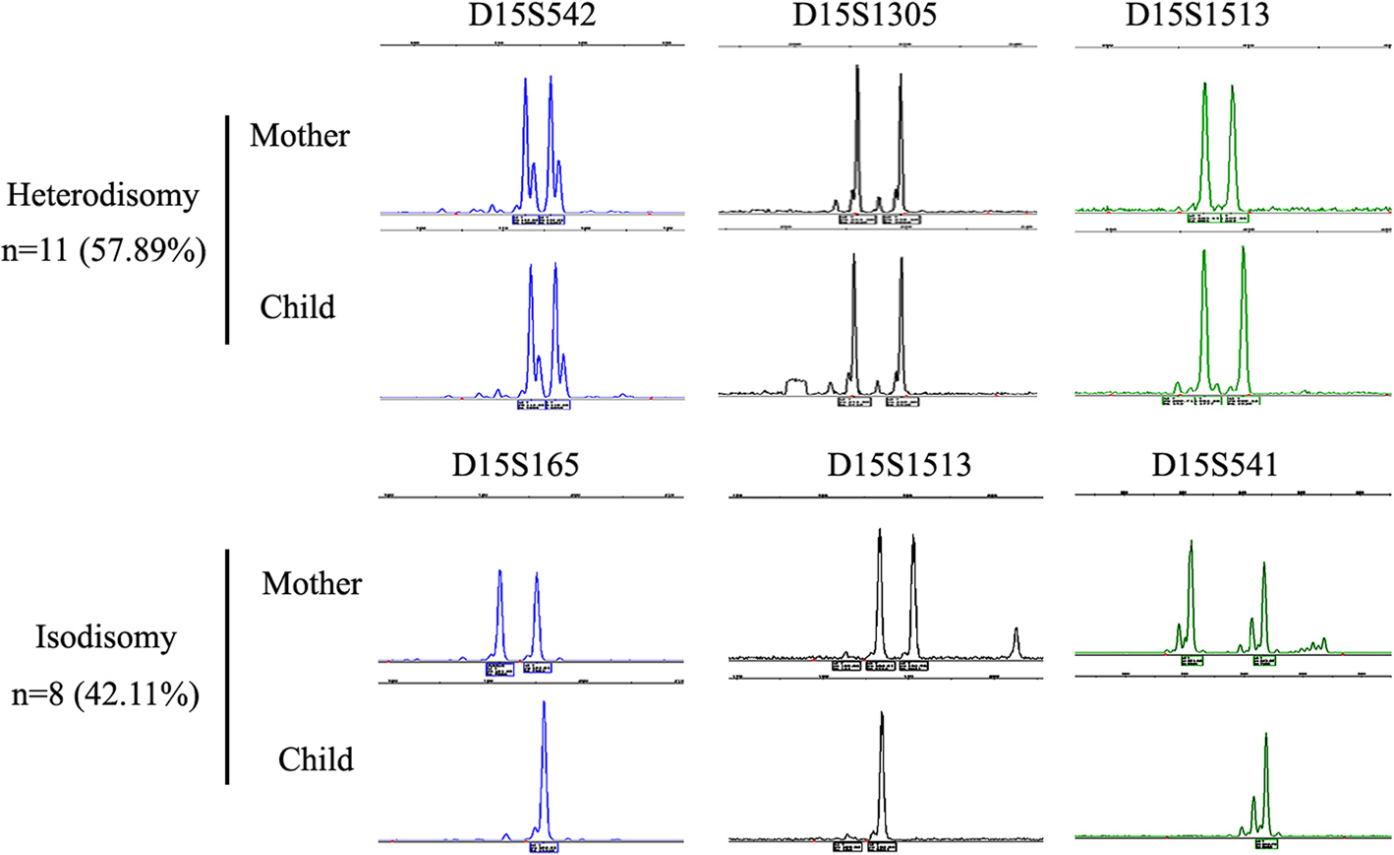
Genetic subtypes of PWS patients with maternal uniparental disomy (UPD). Representative polymorphic microsatellite markers are shown by multiplex fluorescent PCR and capillary electrophoresis

The 19 patients with maternal UPD were subgrouped by genotyping of polymorphic microsatellites using multiplex fluorescent PCR (Fig. 4). Microsatellite markers were informative when heterozygous in the mothers. Those patients with both heterozygous alleles maternally inherited were considered as heterodisomy, and with either maternal allele homozygous were considered as isodisomy. The informative microsatellites of each individual were consistent in inheritance pattern, indicating no crossover events in 15q11-q13 region. Hereby, the 19 PWS patients with maternal UPD were classified into 11 (11/19, 57.89%) heterodisomy and 8 (8/19, 42.11%) isodisomy at 15q11-q13.

### Phenotypic characteristics of 104 patients with different genetic subtypes

The phenotypic information of 104 PWS patients with different genetic subtypes are shown in Table 1. Hypotonia (100%), feeding problems (94.23%), and cryptorchidism (90% in males) were almost universal at neonatal period. Motor delay (100%), short stature (85.29%), and sticky saliva (79.41%) were prevalent since infancy. Learning disabilities (97.59%), speech delay (74.70%), and hyperphagia (73.49%) were common during the childhood. Lack of satiety (86.67%), hypogonadism (90.91%), and incomplete/delayed puberty (81.82%) were frequent in the older patients. The maternal age was significantly higher in the UPD than the deletion group (31.4 ± 3.4 *vs* 27.8 ± 3.8 years, *P* < 0.01). Anxiety (64.29% *vs* 26.09%, *P* < 0.01) and autistic traits (57.14% *vs* 26.09%, *P* < 0.05) were more frequent in the UPD than the deletion group. On the contrary, hypopigmentation (41.18% *vs* 67.95%, *P* < 0.05) and skin picking (42.11% *vs* 69.05%, *P* < 0.05) were more frequent in the deletion than the UPD group. The type I deletion group was diagnosed at earlier age (4.3 ± 4.9 *vs* 6.2 ± 3.2 years, *P* < 0.05) and showed higher frequency of speech delay (95.45% *vs* 63.83%, *P* < 0.05) than the type II deletion group. Although with no statistical significance, a higher frequency of anxiety (83.33% *vs* 50%, *P* = 0.20) was found in the 15q11-q13 isodisomy UPD than heterodisomy UPD group.

**Table 1:** Phenotypic characteristics of 104 patients with different genetic subtypes

	**Parameters**	**Maternal UPD**			**Paternal deletion**		
		**Heterodisomy**	**Isodisomy**	**Total**	**Type I**	**Type II**	**Total**
**General information**	Case evaluated	***n* = 11**	***n* = 8**	***n* = 19**	***n* = 29**	***n* = 56**	***n* = 85**
	Maternal age	32.0 ± 3.5	30.5 ± 3.1	31.4 ± 3.4	28.6 ± 4.5	27.2 ± 3.5	27.8 ± 3.8*
	Diagnosis age						
	≤ 1 year (percentage)	7 (63.64%)	4 (50%)	11 (57.89%)	19 (65.52%)	32 (57.14%)	51 (60.00%)
	> 1 year (average)	4.3 ± 2.5	5.3 ± 3.0	4.8 ± 2.6	3.7 ± 3.3	6.2 ± 3.2^#^	5.6 ± 3.4
	Sex						
	female	5 (45.45%)	3 (37.5%)	8 (42.11%)	12 (42.86%)	24 (42.86%)	36 (42.86%)
	male	6 (54.55%)	5 (62.5%)	11 (57.89%)	17 (58.62%)	32 (57.14%)	49 (57.65%)
**Neonatal (≤ 2 m)**	Cases evaluated	***n* = 11**	***n* = 8**	***n* = 19**	***n* = 29**	***n* = 56**	***n* = 85**
	Hypotonia	11 (100%)	8 (100%)	19 (100%)	29 (100%)	56 (100%)	85 (100%)
	Feeding problems	9 (81.82%)	8 (100%)	17 (89.47%)	29 (100%)	52 (92.86%)	81(95.29%)
	Cryptorchidism (/male)	5/6 (83.33%)	5/5 (100%)	10/11 (90.91%)	15/17 (88.24%)	29/32 (90.63%)	44/49 (89.80%)
	Hypopigmentation	5 (45.45%)	3 (37.5%)	8 (42.11%)	20 (68.97%)	38 (67.86%)	58 (68.24%)*
**Infancy (2 m ~ ≤ 2 y)**	Cases evaluated	***n* = 11**	***n* = 7**	***n* = 18**	***n* = 29**	***n* = 55**	***n* = 84**
	Motor delay	11 (100%)	7 (100%)	18 (100%)	29 (100%)	55 (100%)	84 (100%)
	Dysmorphic face ^a^	4 (36.36%)	2 (28.57%)	6 (33.33%)	12 (41.38%)	20 (36.36%)	32 (38.10%)
	Short stature ^b^	8 (72.73%)	6 (85.71%)	14 (77.78%)	26 (89.66%)	47 (85.45%)	73 (86.90%)
	Small hands and feet	4 (36.36%)	4 (57.14%)	8 (44.44%)	17 (58.62%)	27 (49.09%)	44 (52.38%)
	Sticky saliva	9 (81.82%)	5 (71.43%)	14 (77.78%)	24 (82.76%)	43(78.18%)	67 (79.76%)
	Sleeping disorder	4 (36.36%)	2 (28.57%)	6 (33.33%)	10 (34.48%)	20 (36.36%)	30 (35.71%)
	Temperature instability ^c^	3 (27.27%)	1 (14.29%)	4 (22.22%)	9 (31.03%)	14 (25.45%)	23 (27.38%)
**Childhood (2 y ~ ≤ 10 y)**	Cases evaluated	***n* = 8**	***n* = 6**	***n* = 14**	***n* = 22**	***n* = 47**	***n* = 69**
	Hyperphagia	6 (75%)	4 (66.67%)	10 (71.43%)	17 (77.27%)	34 (72.34%)	51 (73.91%)
	Obesity ^d^	4 (50%)	4 (66.67%)	8 (57.14%)	15 (68.18%)	28 (59.57%)	43 (62.32%)
	Speech delay ^e^	6 (75%)	5 (83.33%)	11 (78.57%)	21 (95.45%)	30 (63.83%)^#^	51 (73.91%)
	Learning disabilities	8 (100%)	6 (100%)	14 (100%)	22 (100%)	45 (95.74%)	67 (97.10%)
	Temper tantrums	5 (62.5%)	4 (66.67%)	9 (64.29%)	16 (72.73%)	31 (65.96%)	47 (68.12%)
	Compulsive behavior	4 (50%)	2 (33.33%)	6 (42.86%)	12 (54.55%)	23 (48.94%)	35 (50.72%)
	Anxiety	4 (50%)	5 (83.33%)	9 (64.29%)	6 (27.27%)	12 (25.53%)	18 (26.09%)*
	Autistic traits	4 (50%)	4 (66.67%)	8 (57.14%)	7 (31.82%)	11 (23.40%)	18 (26.09%)*
	Skin picking	3 (37.5%)	3 (50%)	6 (42.86%)	16 (72.73%)	33 (70.21%)	49 (71.01%)*
	High pain threshold	3 (37.5%)	2 (33.33%)	5 (35.71%)	8 (36.36%)	15 (31.91%)	23 (33.33%)
**Teenage** (**> 10 y)**	Cases evaluated	***n* = 1**	***n* = 1**	***n* = 2**	***n* = 3**	***n* = 10**	***n* = 13**
	Lack of satiety	1 (100%)	1 (100%)	2 (100%)	3 (100%)	8 (80%)	11 (84.62%)
	Cases evaluated > 13y	***n* = 1**	***n* = 0**	***n* = 1**	***n* = 2**	***n* = 8**	***n* = 10**
	Early adrenarche	1 (100%)	NA	1 (100%)	1 (50%)	5 (62.5%)	6 (60%)
	Incomplete/delayed puberty	0 (0%)	NA	0 (0%)	2 (100%)	7 (87.5%)	9 (90%)
	Hypogonadism ^f^	1 (100%)	NA	1 (100%)	2 (100%)	7 (87.5%)	9 (90%)

*NA* Not applicable for evaluation. ^a^Barrow bifrontal diameter, almond-shaped eyes, strabismus, high palates, small chins. ^b^Height < 2 standard deviation score. ^c^Febrile seizures. ^d^BMI > 95 centile. ^e^Less than 30 words at 2 years. ^f^Lack of spontaneous menarche in female or small penis (< 2.5 cm length)/testis (< 4 mL volume) in male. ^*^P < 0.05 of the total deletion group *vs* total UPD group. ^#^*P* < 0.05 of the type II deletion group *vs* type I deletion group

### Phenotypic characteristics of 6 patients with atypical deletions

There were 6 patients with atypical deletions that are smaller than type II or larger than type I. The phenotypic characteristics are listed in Table 2. Patient 1 retained three genes (*MKRN3*, *MAGEL2* and *NDN)* compared with type II patients, and showed cardinal traits of PWS at mild degree. Patient 2 (positive in *NIPA1* and *OCA2*) and patient 3 (positive in *NIPA1*) with smaller deletions than type I showed unremarkable differences to the majority of PWS patients. Hypopigmentation was positive in patient 3 but not in patient 2. Patient 4 and patient 5 had expanded deletions to BP4. Patient 6 with unbalanced chromosomal translocation had a further deletion to BP5. They presented with severe phenotype such as tube feeding in patient 4 and patient 6, and absence of speech in patient 6. No remarkable extra abnormalities were observed in them.

**Table 2:** Phenotypic characteristics of 6 patients with atypical deletions

Parameters	Patient 1	Patient 2	Patient 3	Patient 4	Patient 5	Patient 6
Deletion region	distal BP2–BP3	distal BP1–proximal BP3	distal BP1–BP3	BP1–BP4	BP1–BP4	BP1–BP5
Maternal age	26 years	31 years	28 years	24 years	32 years	25 years
Diagnosis age	< 1 year	3 years	< 1 year	< 1 year	1.5 years	< 1 year
Sex	male	female	male	male	female	male
Neonatal hypotonia	+	+	+	+	+	+
Feeding problems	+	+	+	+	+	+
Cryptorchidism	–	NA	+	+	NA	+
Hypopigmentation	–	–	+	+	+	+
Developmental delay	+	+	+	+	+	+
Dysmorphic face ^a^	–	–	–	–	+	+
Short stature ^b^	+	+	+	+	+	+
Small hands and feet	–	–	–	–	+	+
Sticky saliva	+	+	–	+	+	+
Temperature instability ^c^	–	–	–	+	–	+
Sleeping disorder	–	–	–	–	+	+
Hyperphagia	–	+	+	NA	+	+
Obesity ^d^	–	+	–	NA	+	+
Speech delay ^e^	+	+	+	NA	+	+
Learning disabilities	+	+	+	NA	+	+
Temper tantrums	+	+	+	NA	+	+
Compulsive behavior	–	–	–	NA	+	+
Anxiety	–	–	–	NA	+	+
Autistic trait	–	–	–	NA	+	+
Skin picking	–	+	+	NA	+	+
High pain threshold	–	–	–	NA	–	–
Abnormal karyotype	–	–	–	–	–	+

+ : positive. –: negative, *NA* Not applicable for evaluation. ^a^Almond-shaped eyes, strabismus, narrow bifrontal diameter. ^b^Height < 2 standard deviation score, ^c^Febrile seizures. ^d^BMI > 95 centile. ^e^Less than 30 words at 2 years

### Effect of GH treatment on 21 PWS patients

There were 21 (21/110, 19.09%) patients received recombinant human GH treatment for more than one year. The median age at onset of GH treatment was 2.1 (range 0.9–4.8) years. The anthropometric and laboratory parameters before and after GH treatment are shown in Table 3. There was a significant increase in height SDS (− 0.43 ± 0.68 *vs* − 1.32 ± 1.19, *P* < 0.01) but not in that of weight or BMI. The serum IGF-I SDS was significantly increased after GH treatment (− 0.45 ± 0.48 *vs* − 1.97 ± 1.12, *P* < 0.01). The thyroid function, evaluated from the TSH, fT4, and fT3 levels, was not significantly altered. In addition, no obvious changes were found in parameters of glucose and lipid metabolism.

**Table 3:** Effect of growth hormone (GH) treatment on 21 patients

Parameters	Before GH treatment	After GH treatment	Reference ranges
**General information**			
Case evaluated	*n* = 21		/
Sex: male/female	14/7		/
Genetic subtype: UPD/Del	3/18		/
Median age at onset (range, years)	2.1 (0.9–4.8)		/
**Anthropometry**			
Length/height SDS	– 1.32 ± 1.19	− 0.43 ± 0.68^*^	/
Body weight SDS	0.20 ± 1.76	0.34 ± 1.53	/
BMI SDS	0.75 ± 1.91	1.03 ± 1.65	/
**Laborotory**			
Insulin-like growth factor I (IGF-I) SDS	− 1.97 ± 1.12	− 0.45 ± 0.48^*^	**/**
Thyroid stimulating hormone (TSH, μIU/mL)	1.80 ± 0.86	1.96 ± 0.97	0.30–4.80
Free thyroxine (fT4, pmol/L)	13.02 ± 1.37	11.58 ± 2.04	9.01–19.05
Free triiodothyronine (fT3, pmol/L)	5.52 ± 0.59	5.15 ± 0.80	2.43–6.01
Fasting glucose (mg/dL)	4.96 ± 0.63	4.72 ± 0.77	3.9–6.11
Fasting insulin (μIU/mL)	19.43 ± 8.70	17.43 ± 6.24	3.9–25.00
Total triglyceride (mmol/L)	0.96 ± 0.40	1.14 ± 0.55	0.4–1.69
Total cholesterol (mmol/L)	4.84 ± 1.38	5.02 ± 1.82	3.36–5.69
Low density lipoprotein (LDL, mmol/L)	3.07 ± 1.34	3.88 ± 1.07	< 3.37

^*^P < 0.01

## Discussion

Imprinted genes are regulated by methylation for differential expression according to the parental origin. MS-MLPA has great advantage in the diagnosis of imprinted disorders by detecting both copy number and methylation ratio [6, 21]. In the present study, a high diagnostic rate of 7.70% by ME028 was obtained from 662 individuals with suspected PWS features including neonatal hypotonia with feeding problems, male cryptorchidism, short stature with small hands/feet, obesity with hyperphagia and extreme food seeking. These PWS features deserve high attention of pediatricians, and MS-MLPA was recommended as first-tier molecular technique for rapid diagnosis of PWS. The proportion of maternal UPD was 37.25% in our study, which was higher than the conventional 20–30% [2], and similar to 36.27% in a recent large multisite cohort study [22]. This might be due to higher maternal age nowadays or lower diagnostic rate of UPD previously. In the 7,910 individuals with unexplained DD/ID, MLPA P245 screening was cost-effective to identify multiple microdeletion syndromes at one time. The diagnostic yield for 15q11.2 deletions by P245 was 1.25% in our study, which was lower than 2.07% in 10,026 Chinese pediatric patients with developmental disorders by chromosomal microarray [23], but much higher than 0.14% in 29,085 Western children with unexplained developmental delay by array comparative genomic hybridization [24]. The differences might come from the race, inclusion criteria, or detection methods. The diagnostic rate of paternal deletion PWS was 0.75% by P245-ME028 assays, with the maternal UPD PWS missed out in this group.

The probes of ME028 encompassing BP1 to distal BP3 facilitated the subclassification of paternal deletion PWS. Those atypical deletions exceeding distal BP3 were delineated by CNV-sequencing. The 91 PWS cases with paternal deletions were subdivided into 31.87% of type I, 61.54% of type II and 6.59% of atypical deletions. Limited researches demonstrated the subtypes of paternal deletion PWS. A multisite cohort study including 303 deletion PWS patients (primarily Caucasians) showed 38.9% of type I, 54.5% of type II and 6.6% of atypical deletions [22]. Our data are comparatively lower in type I and higher in type II. More researches in large scale are needed to refine the ratios. PWS with maternal UPD could be subgrouped based on the meiosis errors and crossover events [7, 8]. In the present study by microsatellite genotyping, we found 57.89% of heterodisomy and 42.11% of isodisomy at 15q11-q13 region, corresponding to meiosis I and II errors respectively. This added to the currently less known data about the subtype ratio of PWS with maternal UPD. The mixed form of UPD induced by crossover events at distal 15q was not evaluated.

The clinical characters were evaluated and compared among the PWS genetic subtypes. Overall, the universal phenotype included neonatal hypotonia, feeding problem, motor delay, learning disabilities, and male cryptorchidism. Genital hypoplasia in females was easily overlooked and not assessed. Studies on various populations have compared phenotype between the UPD and deletion types of PWS, showing disparities including maternal age, hypopigmentation, birth length, IQ score, self-injury, psychotic illness, pain threshold and sleeping disorders [25–29]. Our UPD patients had higher maternal age, higher frequencies of anxiety and autistic traits, and lower frequencies of hypopigmentation and skin picking than the deletion patients. Other previously indicated differences were not found including higher diagnosis age in UPD, or more frequent feeding problems, temperature instabilities, sleeping disorders, dysmorphic faces in the deletion group. Our type I patients showed earlier diagnosis age and higher frequency of speech delay, indicating more severe symptoms in response to larger deletion regions than type II. We did not found behavioral differences between type I and type II groups, whereas a previous study on 12 type I and 14 type II PWS individuals showed worse adaptive and more common obsessive–compulsive behaviors in the type I group [29]. Phenotypic variations between heterodisomy and isodisomy in the UPD group are rarely reported. Some individual cases have been reported about the concomitant autosomal recessive disorders of ichthyosis and Tay-Sachs disease caused by pathogenic variants in the homozygosity region [30, 31]. We did not find additional phenotype in the UPD group. A relatively higher frequency of anxiety was observed in the maternal chr 15 isodisomy than heterodisomy group. This has not been reported before, and needs confirmation in more PWS individuals with maternal UPD.

Patients with atypical deletions are informative for understanding the function of distinct deletion regions. Many hallmark traits of PWS were positive in patient 1 retaining the *MKRN3*, *MAGEL2* and *NDN* genes compared with type II patients, indicating the *SNRPN-SNORD* regions were sufficient for PWS pathogenesis. Patient 2 differed to patient 3 in the *OCA2* gene which correlated to the hypopigmentation phenotype. The 15q11.2 BP1–BP2 microdeletion of the *NIPA1*, *NIPA2*, *CYFIP1*, and *TUBGCP5* genes causes Burnside-Butler syndrome with abnormalities in brain morphology, behavior, and cognition [32]. Patient 2 and patient 3 with partial deletion of BP1–BP2 (*NIPA1* retained and *TUBGCP5* deleted) were indistinguishable to the majority of PWS patients. Previous reports of expanded deletion in PWS demonstrated cardiovascular, renal or other congenital anomalies [33]. The three patients with expanded deletions to distal BP4 and BP5 displayed severe phenotype including tube feeding and absence of speech, without structural anomalies.

The benefits of GH treatment have been established on body composition, psychomotor development, cognition, adaptive functioning, and linear growth, without adverse effects on glucose parameters, lipid profile and blood pressure [34]. In the present cohort, 19.09% of PWS patients received GH treatment for more than one year, and with complete laboratory data. We did not fragment these patients into subtypes. Significant improvements were found in body height and serum IGF-I. Central hypothyroidism has been found in very young patients with PWS, and improves with age [35]. Our data of TSH, fT4, and fT3 were within reference ranges before and after GH treatment, with no obvious changes. The glucose and lipid metabolic parameters were also within reference ranges. These possibly due to the relatively early nutritional phases of the patients.

## Conclusion

This study provided detailed genetic and phenotypic characters of 110 PWS patients diagnosed from 8,572 Chinese pediatric individuals. We added more data about the phenotypic associations with various genetic subtypes, which help to promote awareness of this complex neurodevelopmental disorder.

## Supplementary Information

**Additional file 1: Table S1.** Primers for microsatellite markers.

## Data Availability

The data set used in the current study can be available from the corresponding author on reasonable request.

## Abbreviations

PWS: Prader-Willi syndrome
UPD: Uniparental disomy
GH: Growth hormone
SDS: Standard deviation score
BP: Breakpoint
DD/ID: Developmental delay and/or intellectual disability
DQ: Development quotient
IQ: Intelligent quotient
IGF-I: Insulin-like growth factor I
TSH: Thyroid stimulating hormone
fT4: Free thyroxine
fT3: Free triiodothyronine
LDL: Low density lipoprotein
MLPA: Multiplex ligation-dependent probe amplification
MS-MLPA: Methylation-specific MLPA

## References

[1] Cassidy S, Schwartz S, Miller JL, Driscoll DJ (2012). Prader-Willi syndrome. Genet Med.

[2] Angulo MA, Butler MG, Cataletto ME (2015). Prader-Willi syndrome: a review of clinical, genetic, and endocrine findings. J Endocrinol Invest.

[3] Pujana MA, Nadal M, Guitart M, Armengol L, Gratacòs M, Estivill X (2002). Human chromosome 15q11-q14 regions of rearrangements contain clusters of LCR15 duplicons. Eur J Hum Genet.

[4] Amos-Landgraf JM, Ji Y, Gottlieb W, Depinet T, Wandstrat AE, Cassidy SB (1999). Chromosome breakage in the Prader-Willi and Angelman syndromes involves recombination between large, transcribed repeats at proximal and distal breakpoints. Am J Hum Genet.

[5] Kim SJ, Miller JL, Kuipers PJ, German JR, Beaudet AL, Sahoo T, Driscoll DJ (2012). Unique and atypical deletions in Prader-Willi syndrome reveal distinct phenotypes. Eur J Hum Genet.

[6] Henkhaus RS, Kim SJ, Kimonis VE, Gold JA, Dykens EM, Driscoll DJ, Butler MG (2012). Methylation-specific multiplex ligation-dependent probe amplification and identification of deletion genetic subtypes in Prader-Willi syndrome. Genet Test Mol Biomarkers.

[7] Robinson WP (2000). Mechanisms leading to uniparental disomy and their clinical consequences. BioEssays.

[8] Horsthemke B, Wagstaff J (2008). Mechanisms of imprinting of the Prader-Willi/Angelman region. Am J Med Genet A.

[9] Çizmecioğlu FM, Jones JH, Paterson WF, Kherra S, Kourime M, McGowan R (2018). Neonatal Features of the Prader-Willi Syndrome; The Case for Making the Diagnosis During the First Week of Life. J Clin Res Pediatr Endocrinol.

[10] Miller JL, Lynn CH, Driscoll DC, Goldstone AP, Gold JA, Kimonis V (2011). Nutritional phases in Prader-Willi syndrome. Am J Med Genet A.

[11] Dykens EM, Roof E (2008). Behavior in Prader-Willi syndrome: relationship to genetic subtypes and age. J Child Psychol Psychiatry.

[12] Burman P, Ritzén EM, Lindgren AC (2001). Endocrine dysfunction in Prader-Willi syndrome: a review with special reference to GH. Endocr Rev.

[13] Swaab DF (1997). Prader-Willi syndrome and the hypothalamus. Acta Paediatr Suppl.

[14] Ball RS (1977). The Gesell Developmental Schedules: Arnold Gesell (1880–1961). J Abnorm Child Psychol.

[15] Wechsler D (2014). Wechsler Intelligence Scale for Children.

[16] Schopler E, Reichler RJ, DeVellis RF, Daly K (1980). Toward objective classification of childhood autism: Childhood Autism Rating Scale (CARS). J Autism Dev Disord.

[17] Li H, Ji CY, Zong XN, Zhang YQ (2009). Height and weight standardized growth charts for Chinese children and adolescents aged 0 to 18 years. Zhonghua Er Ke Za Zhi.

[18] Li H, Ji CY, Zong XN, Zhang YQ (2009). Body mass index growth curves for Chinese children and adolescents aged 0 to 18 years. Zhonghua Er Ke Za Zhi.

[19] Tsai WY, Fang LJ, Lee JS (1993). Concentrations of serum insulin-like growth factor-I (IGF-I) in normal Chinese children and children with growth hormone deficiency. J Formos Med Assoc.

[20] Liang D, Peng Y, Lv W, Deng L, Zhang Y, Li H (2014). Copy number variation sequencing for comprehensive diagnosis of chromosome disease syndromes. J Mol Diagn.

[21] Bittel DC, Kibiryeva N, Butler MG (2007). Methylation-specific multiplex ligation-dependent probe amplification analysis of subjects with chromosome 15 abnormalities. Genet Test.

[22] Butler MG, Hartin SN, Hossain WA, Manzardo AM, Kimonis V, Dykens E (2019). Molecular genetic classification in Prader-Willi syndrome: a multisite cohort study. J Med Genet.

[23] Yuan H, Shangguan S, Li Z, Luo J, Su J, Yao R (2021). CNV profiles of Chinese pediatric patients with developmental disorders. Genet Med.

[24] Coe BP, Witherspoon K, Rosenfeld JA, van Bon BW, Vulto-van Silfhout AT, Bosco P (2014). Refining analyses of copy number variation identifies specific genes associated with developmental delay. Nat Genet.

[25] Varela MC, Kok F, Setian N, Kim CA, Koiffmann CP (2005). Impact of molecular mechanisms, including deletion size, on Prader-Willi syndrome phenotype: study of 75 patients. Clin Genet.

[26] Fridman C, Varela MC, Kok F, Setian N, Koiffmann CP (2000). Prader-Willi syndrome: genetic tests and clinical findings. Genet Test.

[27] Roof E, Stone W, MacLean W, Feurer ID, Thompson T, Butler MG (2000). Intellectual characteristics of Prader-Willi syndrome: comparison of genetic subtypes. J Intellect Disabil Res.

[28] Dykens EM, Cassidy SB, King BH (1999). Maladaptive behavior differences in Prader-Willi syndrome due to paternal deletions versus maternal uniparental disomy. Am J Ment Retard.

[29] Butler MG, Bittel DC, Kibiryeva N, Talebizadeh Z, Thompson T (2004). Behavioral differences among subjects with Prader-Willi syndrome and type I or type II deletion and maternal disomy. Pediatrics.

[30] Muthusamy K, Macke EL, Klee EW, Tebben PJ, Hand JL, Hasadsri L (2020). Congenital ichthyosis in Prader-Willi syndrome associated with maternal chromosome 15 uniparental disomy: Case report and review of autosomal recessive conditions unmasked by UPD. Am J Med Genet A.

[31] Regier DS, Leon E, Counts DR, Tifft CJ, Zand DJ (2015). Concurrent diagnoses of Prader-Willi syndrome and GM2 gangliosidosis caused by uniparental disomy of chromosome 15. Am J Med Genet A.

[32] Rafi SK, Butler MG (2020). The 15q11.2 BP1-BP2 Microdeletion (Burnside-Butler) Syndrome: Silico Analyses of the Four Coding Genes Reveal Functional Associations with Neurodevelopmental Phenotypes. Int J Mol Sci.

[33] Liu AP, Tang WF, Lau ET, Chan KY, Kan AS, Wong KY (2013). Expanded Prader-Willi syndrome due to chromosome 15q11.2–14 deletion: report and a review of literature. Am J Med Genet A.

[34] Bakker NE, Kuppens RJ, Siemensma EP, Tummers-de van Wijngaarden RF, Festen DA, Bindels-de Heus GC (2013). Eight years of growth hormone treatment in children with Prader-Willi syndrome: maintaining the positive effects. J Clin Endocrinol Metab..

[35] Konishi A, Ida S, Shoji Y, Etani Y, Kawai M (2021). Central hypothyroidism improves with age in very young children with Prader-Willi syndrome. Clin Endocrinol (Oxf).

