# Supplementary material for: Genetic subtypes and phenotypic characteristics of 110 patients with Prader-Willi syndrome
Source: Ital J Pediatr. 2022 Jul 23;48:121. doi: 10.1186/s13052-022-01319-1 (PMC9308266; doi:10.1186/s13052-022-01319-1)
Supplement: Supplementary file 1 — Additional file 1: Table S1. Primers for microsatellite markers. [file 13052_2022_1319_MOESM1_ESM.doc]

**Supplementary Table 1** Primers for microsatellite markers

| **Markers** | **Break points (BPs)** | **Forward primer** | **Reverse primer** | **Fluorescence** |
| --- | --- | --- | --- | --- |
| D15S541 | BP1-BP2 | GCATTTTTGGTTACCTGTATG | GTCTTCCAGGTTTATGGTTGTC | TAMRA |
| D15S542 |  | AGCAGACTCCGGAACCTCATC | CCTGCCTTCTTGCTGGGGCTG | 6-FAM |
| D15S1035 | BP2-BP3 | AGCAGACTCCGCAACCTCA | GGAAACTGCCCTGCTGG | TAMRA |
| D15S11 |  | AGGCATAACTGCATAGTAAATG | CAGGCATCCATTTTGAATAGAG | HEX |
| D15S128 | BP3-BP4 | CATTGCATTTGTATGCAGC | TCTGTTTTCCTTGCCTGAG | 6-FAM |
| D15S1513 |  | GGATAAGAAGGATAAAAGTCC | GAATAAACTTGACATCCTCC | HEX |
| D15S1043 | BP4-BP5 | GAACTATGAGGCAAGGAAC | AGAAACCGTGATGTAGGG | 6-FAM |
| D15S1048 |  | AATGAGCCGTCTTTGTGCC | AGAAAGGAGCAAGTGTTGGT | TAMRA |
| D15S165 | Distal BP5 | GTTTACGCCTCATGGATTTA | GGGCACACAGTCCCAA | 6-FAM |
| D15S123 |  | TGAACCCAATGGACTCCTG | TTCATGCCACCAACAAACTT | HEX |
